# A Novel Arthroscopic Technique for Intraoperative Mobilization of Synovial Mesenchymal Stem Cells

**DOI:** 10.1177/0363546518803757

**Published:** 2018-11-12

**Authors:** Thomas G. Baboolal, Alam Khalil-Khan, Anthony A. Theodorides, Owen Wall, Elena Jones, Dennis McGonagle

**Affiliations:** † Faculty of Medicine, Leeds Institute of Rheumatic and Musculoskeletal Medicine, University of Leeds, Leeds, West Yorkshire, UK; ‡ NIHR Leeds Biomedical Research Centre, Chapel Allerton Hospital, Leeds Teaching Hospital Trust, Leeds, West Yorkshire, UK; § Chapel Allerton Orthopaedic Centre, Chapel Allerton Hospital, Leeds Teaching Hospital Trust, Leeds, West Yorkshire, UK; Investigation performed at Chapel Allerton Orthopaedic Centre, Leeds, UK, and the Leeds Institute of Rheumatic and Musculoskeletal Medicine, University of Leeds, Leeds, UK

**Keywords:** mesenchymal stem cells, synovium, synovium-derived stem cell, minimally manipulated, single-stage procedure

## Abstract

**Background:**

Mesenchymal stem cells (MSCs) have emerged as a promising candidate for tissue regeneration and restoration of intra-articular structures such as cartilage, ligaments, and menisci. However, the routine use of MSCs is limited in part by their low numbers and the need for methods and procedures outside of the joint or surgical field.

**Purpose:**

To demonstrate feasibility of a technique in which minimally manipulated synovial MSCs can be mobilized during knee arthroscopy, thereby showing proof of concept for the future evaluation and clinical use of native joint resident MSCs in single-stage joint repair strategies.

**Study Design:**

Descriptive laboratory study.

**Methods:**

Patients (n = 15) undergoing knee arthroscopy who were free from synovitis or active inflammation were selected. Three samples of irrigation fluid were collected from each patient at inception of the procedure, after an initial inspection of the joint, and after agitation of the synovium. MSC numbers were evaluated by colony forming unit–fibroblastic assay. The phenotype of synovial fluid resident and synovial-mobilized MSCs was determined by flow cytometry, and their functionality was determined by trilineage differentiation. Adhesion of culture-expanded mobilized MSCs to fibrin scaffolds was also evaluated to ascertain whether mobilized MSCs might concentrate at sites of bleeding.

**Results:**

Normal irrigation during arthroscopy depleted resident synovial fluid MSCs (4-fold decrease, n = 15). Numbers of MSCs mobilized through use of a purpose-made device were significantly higher (105-fold) than those mobilized through use of a cytology brush (median of 5763 and 54 colonies, respectively; *P* = .001; n = 15). The mobilized cellular fraction contained viable MSCs with proliferative potential and trilineage differentiation capacity for bone, cartilage, and fat lineages, and cultured daughter cells exhibited the standard MSC phenotype. Following culture, mobilized synovial MSCs also adhered to various fibrin scaffolds in vitro. The technique was simple and convenient to use and was not associated with any complications.

**Conclusion:**

Numbers of functional MSCs can be greatly increased during arthroscopy through use of this technique to mobilize cells from the synovium.

**Clinical Relevance:**

This study highlights a novel, single-stage technique to increase joint-specific, synovial-derived MSCs and thereby increase the repair potential of the joint. This technique can be undertaken during many arthroscopic procedures, and it supports the principle of integrating mobilized MSCs into microfracture sites and sites of bleeding or targeted repair through use of fibrin-based and other scaffolds.

Articular cartilage is essential for synovial joint function, providing a low-friction surface to allow joint movement. The repair of cartilage, however, is limited given its avascular, aneural, and alymphatic nature. Consequently, management of isolated articular cartilage defects poses numerous challenges, and these defects, if left untreated, are risk factors for developing osteoarthritis (OA) in later life.^9^ For more advanced stages of OA, management of the disease comes at a high global and socioeconomic burden secondary to the effects of disability, comorbid disease, and expense of treatment.^5^ As such, more effective, earlier treatments are needed.

Advanced treatment of cartilage defects is available in the form of autologous chondrocyte implantation (ACI). However, ACI is a 2-stage procedure; the first stage requiring harvesting of healthy tissue followed by tissue processing and cellular expansion outside of the surgical field, and the second stage entails reimplantation into the defect site. However, long-term follow-up results of ACI are no better than those of microfracture, a more conservative, much less expensive, single-stage procedure.^6,23^ Mesenchymal stem cells (MSCs) have been proposed as an alternative source of reparative cell owing to their ease of harvest and their proliferative and differentiation capacity.^36,37^ MSCs are multipotent and are internationally recognized by their characteristic adherence to plastic; colony-forming capacity; expression of cell surface markers CD73, CD90, and CD105; lack of expression of CD14, CD34, CD45, and HLA-DR; and ability to differentiate in vitro into osteoblasts, chondrocytes, and adipocytes.^10^ Several clinical studies using MSCs to treat OA^24,25,34,43,44,46^ have shown positive results; however, similar to ACI, many MSC methods incorporate tissue or cell extraction (from either bone marrow or adipose tissue) before culture expansion and reimplantation. Intra-articular injection of culture-expanded MSCs has also been hampered by both cost and loss of potency.^21,40^ Other strategies being explored include the combination of various techniques such as scaffolds and fibrin glue for implantation and retention at sites of injury, which appeared promising at second-look arthroscopy.^21,22,45^

A major drawback with the aforementioned strategies involving cellular therapy is the need for more than one operative procedure. Recent single-stage strategies for joint repair include the use of adipose tissue stromal vascular fraction, which contains MSCs.^35^ However, for single-stage cartilage repair and OA, joint cavity MSCs including synovium and synovial fluid (SF) are of particular interest as these populations of MSCs have superior chondrogenic capacity and may be more readily and conveniently manipulated since they are already in the surgical field.^30,32,39^ It is now recognized that the knee joint cavity has comparatively abundant reservoirs of MSCs,^30^ and such MSCs might play a role in joint homeostasis and repair. These MSCs are naturally shed from the synovium^20^ and are elevated with joint injury and early OA,^18,41^ but their repair capacity may be limited due to interactions with SF hyaluronan that limit their adhesion to cartilage.^4^ Current arthroscopic procedures may inadvertently remove these cells (via saline irrigation), and procedures such as microfracture to encourage MSCs to percolate from the bone marrow may be inefficient as a result of low and variable numbers of MSCs in bone marrow.^13,29^

In this study, we hypothesized that arthroscopic procedures likely wash away resident SF-MSCs imbued with reparative potential (Figure 1). Our principle aim was to report the feasibility of a technique that entails a simple, single-stage procedure whereby MSCs are dislodged (mobilized) from the synovium, thereby increasing numbers of joint cavity MSCs with access to cartilage and potentially increasing the reparative potential of the joint during a range of arthroscopic procedures. We sought to determine whether these synovial-mobilized MSCs (Sm-MSCs) would preserve their in vitro MSC phenotype and function. Finally, we asked whether Sm-MSCs would rapidly adhere to blood clots and fibrin scaffolds (in vitro) as a surrogate for what could happen in vivo after microfracture or use of a fibrin glue.

**Figure 1.**
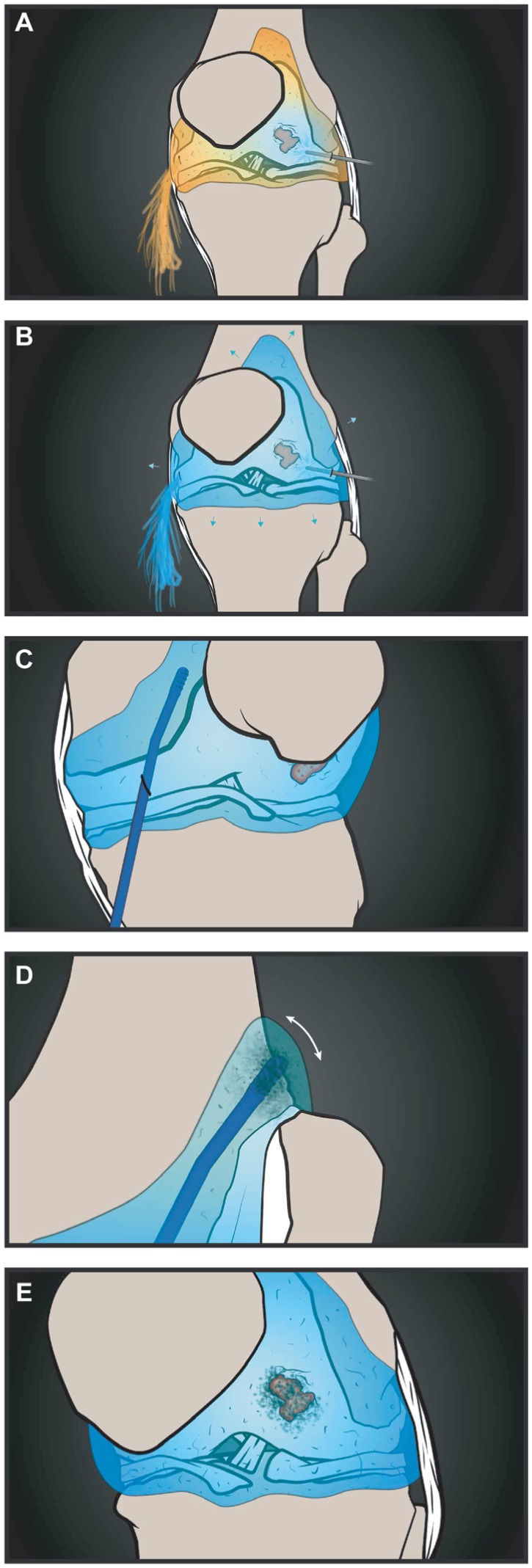
A proposed use of the technique to mobilize synovial mesenchymal stem cells (MSCs) and supplement cartilage healing. (A, B) Joint irrigation replaces synovial fluid (SF), leading to a loss of resident SF-MSCs while inflating the knee. (C, D) The stem cell mobilizing device (STEM device) is introduced into the joint cavity and maneuvered into the suprapatellar pouch where it physically dislodges cells, including MSCs, from the synovial lining. (E) These MSCs are released into the cavity, where they may supplement those from the bone marrow and participate in repair at sites such as cartilage defects via adhesion to the clot after microfracture.

## Methods

### Patient Recruitment

Patients included in the study were treated arthroscopically for a spectrum of knee conditions (Table 1) including ligament, meniscal, and cartilage injury, reflecting the varied nature of current practice and procedures that may warrant stem cell treatment. The average age of recruited patients was 32 years, ranging between 18 and 53 (n = 15). At the time of arthroscopy, patients were free from joint effusion, synovitis, or active inflammation as determined during arthroscopy. Ethical approval for this study was granted by the UK Research Ethics Council, and all study participants were recruited after providing informed written consent.

**Table 1:** Study Participant (n = 15) Characteristics and Arthroscopic Procedure

Age, y	Sex	Surgery
25.4	F	Lateral meniscal tear repair
39.0	M	Anterior cruciate ligament reconstruction
18.1	M	Lateral meniscal tear repair and anterior cruciate ligament reconstruction
19.3	M	Lateral meniscal tear repair
19.9	F	Lateral partial meniscal tear repair
52.0	M	Medial partial meniscal tear repair
29.5	M	Anterior cruciate ligament reconstruction
41.3	M	Medial osteochondral fragment and medical meniscal tear repair^*a*^
26.2	M	Medial meniscal tear repair and anterior cruciate ligament reconstruction^*a*^
45.3	F	Medial chondroplasty^*a*^
27.1	M	Medial chondroplasty^*a*^
22.0	M	Partial lateral meniscal tear repair^*a*^
53.7	M	Medial chondroplasty and medical meniscal tear repair^*a*^
25.4	M	Anterior cruciate ligament reconstruction and medial meniscal tear repair^*a*^
40.3	M	Lateral collateral ligament rupture repair^*a*^

a Denotes participants undergoing mesenchymal stem cell mobilization with the stem cell mobilizing device (STEM device).

### Retrieval of MSCs From Irrigation Fluid

Normal saline was used to inflate the knee joint to adequately visualize all joint compartments. To achieve this, gravity-fed saline was irrigated through the capsule to maintain a sufficient intra-articular pressure to obtain arthroscopic views. Irrigation fluid was collected from each patient at 3 stages during knee arthroscopy. The first sample contained resident SF-MSCs and was collected by aspirating the initial saline used to irrigate the joint cavity (5-50 mL collected; mean, 38 mL). A second sample of fluid was then collected after inspection of the knee compartments to evaluate the extent to which native SF-MSCs were being “washed away” during surgery (25-50 mL collected; mean, 39 mL). This sample was collected before any procedure that breached the subchondral bone, in settings where this was undertaken. Each sample was placed into a sterile container before transport to the laboratory.

### Mobilization of Synovial MSCs

**Figure 2.**
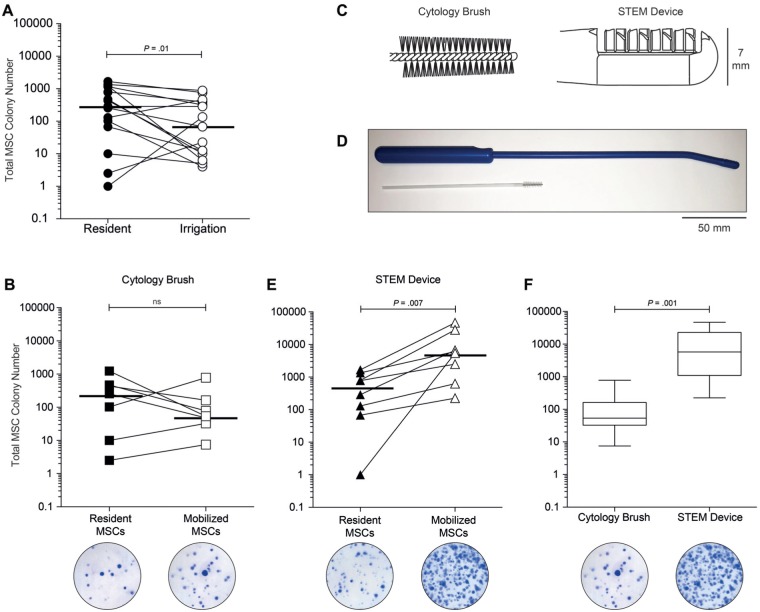
Numbers of mesenchymal stem cells (MSCs) can be increased during arthroscopy by agitation of the synovium. (A) MSC colony number declines during arthroscopy due to loss of resident cells after saline irrigation (n = 15, horizontal bars represent medians). (B) MSC colony numbers can be recovered after mobilization from the synovium by use of a standard cytology brush (horizontal bars represent medians, n = 7) (below, example of representative colony forming unit–fibroblastic [CFU-F] dishes). (C) Comparison of the head design and abrasive surfaces of the cytology brush and the purpose-made stem cell mobilizing device (STEM device). The rigidity of the STEM device, the bullet-shaped nose, and placement of the projections enable easier insertion into the knee through the soft tissues. (D) Comparison of the length and overall design between the cytology brush and the STEM device (C and D to scale). The increased length and angled head allowed for the STEM device to be maneuvered into the suprapatellar pouch. (E) MSC colony number is greatly increased after use of the STEM device during arthroscopy (horizontal bars represent medians, n = 8) (below, example of representative CFU-F dishes). (F) Colony numbers are significantly increased (>100-fold) by use of the STEM device compared with the cytology brush (n = 15) (below, example of representative CFU-F dishes). ns, nonsignificant.

The feasibility of mobilizing MSCs from the synovium during arthroscopy was investigated by collecting a third sample of irrigation fluid after agitation of the synovium to mobilize MSCs. To do this we used a cytology brush (Figure 2C) routinely used for diagnostic purposes to sample cells from mucosal surfaces through natural orifices. Before the induction of the cytology brush, saline irrigation was stopped, leaving sufficient saline to distend the joint cavity. The cytology brush (length, 180 mm; diameter of nylon bristled brush head, 7 mm) (Medical Wires and Equipment Ltd) was then introduced into the joint through the medial portal, under arthroscopic guidance, and the synovial surface of the medial gutter was agitated using the bristled portion for 1 minute. Mobilized cells were collected by opening the outflow valve of the arthroscope. Samples (n = 7; 50-70 mL; mean, 53 mL) were collected in a sterile container before transportation to the laboratory.

The cytology brush was difficult to introduce into the knee portal due to its inherent flexibility and was limited in the range of synovium that could be agitated. To further improve the capability to mobilize synovial MSCs, we used a purpose-made device fabricated from medical-grade acetal copolymer, the stem cell mobilizing device (STEM device) (Figure 2, D and E). Similar to the cytology brush, this optimized device was sterile, was intended for single-use, and was introduced through the medial knee portal. The STEM device was tailored for arthroscopic use with a stiffer arm and blunted end to aid insertion and to limit potential inadvertent damage to other joint structures. The design also allowed the STEM device to be guided into the suprapatellar pouch, where a larger area of fibrous synovium could be agitated to maximize MSC mobilization. As described above, cells were mobilized by agitating the bristled portion on the synovial surface for 1 minute. Cells were retrieved by irrigation and aspiration of saline from the joint (n = 8; 50-70 mL; mean, 53 mL).

### Retrospective Clinical Follow-up

Clinical data for all patients receiving synovial agitation via the STEM device were evaluated retrospectively by a senior soft tissue knee fellow (A.A.T.). At the time, this fellow had completed training and had passed the FRCS examination. The surgeon gave his opinion regarding the likely effect that mobilizing MSCs would have on the expected progression of each participant for his or her indicated procedure (Table 1). Clinical follow-up duration ranged from 6 weeks to 1 year.

### In Vitro Growth and Quantification of MSC Colony Number

Colony forming unit–fibroblastic (CFU-F) assays were used to determine the number of viable MSCs in each sample. In brief, samples of joint irrigate were centrifuged to pellet cells before resuspending in 10 mL of StemMACS expansion media (Miltenyi Biotec). Duplicate 100 mm–diameter dishes (containing 2 mL of the cell suspension) for each sample were cultured for 14 days in StemMACS expansion medium with twice-weekly media changes, before both dishes were fixed in 3.7% formalin and stained with 1% methylene blue (Sigma-Aldrich). All colonies containing more than 50 cells (verified under a microscope) were counted. The remaining sample was either expanded for further assays or frozen. For expansion, cells were cultured in StemMACS expansion medium with twice-weekly media changes until cells reached approximately 90% confluency before being passaged. MSCs were expanded for 3 or 4 passages, all cells were incubated at 37°C and 5% CO_2_.

### Immunophenotype of MSCs

Flow cytometry was performed on matched, culture-expanded SF-MSCs and Sm-MSCs (n = 3 donors) using a standard panel of markers to define cultured MSCs^10^ through use of an LSRII 4-laser flow cytometer (BD Biosciences). The following antibodies and appropriate isotype controls were used: anti-CD34-allophycocyanin-cyanine (APC), anti-CD19-phycoerythrin (PE), anti-CD45-phycoerythrin-cyaine (PE-Cy7), anti-CD14-fluorescein isothiocyanate (FITC), anti-CD73-PE, anti-CD90-PE-Cy7, and anti-CD105-PE (all from BD Biosciences). Dead cells were discriminated by use of 4′,6-diamidino-2-phenylindole (DAPI; Sigma-Aldrich). At least 10,000 live cell events were collected for each antibody combination.

### Trilineage Differentiation of MSCs

Trilineage differentiation of donor-matched, culture-expanded SF-MSCs and Sm-MSCs (n = 5 donors) was performed by use of standard protocols.^2,17,19,36^ Osteogenic differentiation was assessed with alkaline phosphatase and Alizarin red staining on days 14 and 21 of culture, respectively. The medium used for osteogenic differentiation contained Dulbecco’s modified eagle medium (DMEM) and 100 mg/mL penicillin-streptomycin (Gibco); 10% fetal calf serum (FCS) (Biosera); and 100 nM dexamethasone, 10 mM β-glycerolphosphate, and 50 µM ascorbic acid (all from Sigma-Aldrich). Quantitative analysis of calcium deposition was performed on day 21 according to the manufacturer’s instructions (calcium assay; Senitial Diagnostics). Adipogenic differentiation was assessed with Oil Red-O staining and quantified using Nile red fluorescence on day 21 of culture. The medium used for adipogenic differentiation contained DMEM and 100 mg/mL penicillin-streptomycin (Gibco); 10% FCS (Biosera); 10% horse serum (Stem Cell Technologies); and 500 µM hydrocortisone, 500 µM isobutylmethylxanthine, and 60 µM indomethacin (all from Sigma-Aldrich). Pellet cultures were used to evaluate chondrogenesis on day 21 of culture; the medium contained high-glucose DMEM supplemented with 100 mg/mL penicillin-streptomycin and 100 µg/mL sodium pyruvate, 40 µg/mL L-proline, 50 µg/mL L-ascorbic acid 2-phosphate, 1.25 mg/mL bovine serum albumin, ITS+ (a 1× mixture of recombinant human insulin, human transferrin, and sodium selenite), 100 nM dexamethasone, and 10 ng/mL recombinant human transforming growth factor-β3 (all from Sigma-Aldrich). For quantitative analysis, glycosaminoglycan production was measured according to the manufacturer’s instructions (Blyscan assay; Biocolor). Toluidine blue staining was used for qualitative analysis of frozen sections.

### MSC Adhesion to Biological Scaffolds

Culture-expanded Sm-MSCs were used to assess their ability to adhere to various biological scaffolds. Scaffolds were formed from whole blood (WB), platelet-rich plasma (PRP), or fibrin glue (FG). Blood from healthy volunteers was collected in sodium citrate tubes. PRP was prepared after centrifugation at 250*g* for 10 minutes. Platelets were further concentrated from the supernatant by centrifugation at 1500*g* for 10 minutes, and the pelleted platelets were resuspended in one-fifth of their original volume using donor matched serum. FG scaffolds were formed with bovine purified fibrinogen (23.7 mg/mL) resuspended in StemMACS expansion media. All scaffolds including WB were formed after coagulation by the addition of 100 mM calcium chloride (CaCL_2_) and 50 U/mL thrombin (Sigma-Aldrich). To each scaffold and each time point (in triplicate), 10,000 Sm-MSCs were added and allowed to adhere for 10, 30, or 60 minutes before the supernatant was removed and any nonadherent cells were transferred to tissue culture well. These cells were allowed to attach to the culture well before fixing, staining with 1% methylene blue, and counting. A standard curve using a known number of cells was used to interpolate counted cells as a percentage of initial cell number.

### Statistical Analysis

Due to the limited number of samples, all data were assumed to be nonparametric. As such, paired samples were analyzed with Wilcoxon single rank test and nonpaired samples with Mann-Whitney *U* test. The confidence level for each was set at 95%. All statistical analysis was performed with SPSS version 21 (IBM).

## Results

### Retrieval and Enumeration of MSCs From Irrigation Fluid

CFU-F numbers (a measure of viable MSCs) varied between donors (Figure 2A), with MSC colony number significantly greater (*P* = .01, n = 15) in the initial irrigate (median, 360; range, 1-1675) compared with the second sample (median, 68; range, 4-885). Samples of subsequent irrigation fluid indicated that MSC numbers on average decreased 4-fold over the course of surgery, suggesting that standard orthopaedic practice depletes the joint of stem cells.

### Intraoperative Mobilization of MSCs From the Synovium

Resident SF-MSCs were collected as above, and these served as a baseline to assess the ability of the cytology brush to mobilize MSCs from the synovium. The number of resident SF-MSCs in these donors (n = 7) ranged from 3 to 1235 (Figure 2B). After synovial agitation with the cytology brush, in comparison with MSC numbers obtained before synovial mobilization, the MSC colony increased approximately 5-fold. However, MSC numbers declined relative to the baseline resident SF population from the initial irrigate, ranging from 8 to 775. The median number of colonies formed decreased from 256 in the resident population to 54 after synovial agitation, representing a 3.7-fold decrease (not significant).

### MSCs Are Further Augmented Using a Purpose Made Device

Intraoperative use of the cytology brush mobilized low numbers of synovial lining MSCs. This could be attributable to the limited area of synovium that the cytology brush could access and because this type of device is intended to trap and remove cells from the body. To overcome this limitation and to further increase MSC number during surgery, we used a purpose-made device (the STEM device; Figure 2, C and D) to ascertain whether this pool of regenerative cells located in the synovial lining could be further exploited. In each case (n = 8), the use of the STEM device reliably and significantly increased the number of MSCs mobilized intraoperatively over those resident in the SF (Figure 2E). After synovial agitation with the STEM device, the median number of colonies formed was 5763 (range, 225-46,500), representing a 10-fold increase over the matched SF resident population (*P* = .007; median, 531; range, 1-1675). In comparison with the cytology brush, the STEM device mobilized 105-fold greater viable MSCs from the synovial lining during arthroscopy (Figure 2F) (*P* = .001). Finally, we retrospectively examined follow-up clinical data for those patients who received MSC mobilization with the STEM device. From these data, none of the patients appeared to fare worse than would be expected for their primary surgery had they not undergone MSC mobilization with this device.

### Mobilized MSCs Are Phenotypically and Functionally Comparable to SF-MSCs

**Figure 3.**
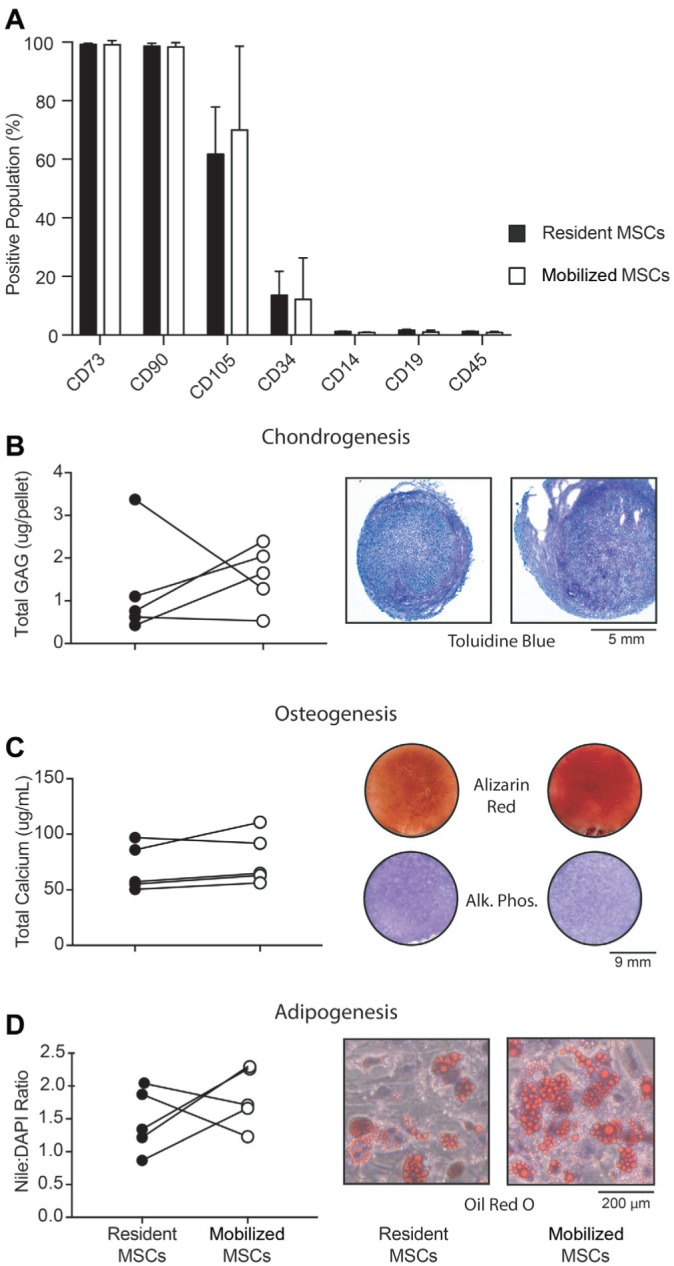
Mobilized mesenchymal stem cells (MSCs) are indistinguishable from resident synovial fluid (SF)–MSCs. (A) Resident and mobilized MSCs have a comparable immunophenotype, which is consistent with both SF- and synovium-derived MSCs (n = 3 matched donors). (B-D) Resident and mobilized MSCs have comparable trilineage differentiation capacity as demonstrated by quantitative assays (left, n = 5 matched donors) illustrating their chondrogenic, osteogenic, and adipogenic potential, respectively, and qualitative assays (right, representative matched donors).

To determine whether Sm-MSCs were comparable to their resident SF counterparts and withstood the biophysical manipulation, we used standard techniques on culture-expanded, donor-matched cells to perform immunophenotyping and multilineage differentiation. Both SF-MSCs and Sm-MSCs were positive for standard MSCs surface markers CD73, CD90, and CD105 and negative for common hematopoietic lineage markers CD14, CD19, CD45, and CD34 (Figure 3A), confirming the feasibility of this procedure to mobilize stem cells during the surgical procedure.

Differentiation of Sm-MSCs toward chondrogenic (Figure 3B), osteogenic (Figure 3C), and adipogenic (Figure 3D) lineages was also compared with donor-matched SF-MSCs. In each case, qualitative and quantitative assay demonstrated that the differentiation capacities of the mobilized MSCs were comparable to those of their matched SF counterparts. These data show that Sm-MSCs are comparable to SF-MSCs, consistent with previous literature describing synovium as a source of intra-articular MSCs.^39^

### Mobilized MSCs Rapidly Adhere to Biological Scaffolds

**Figure 4.**
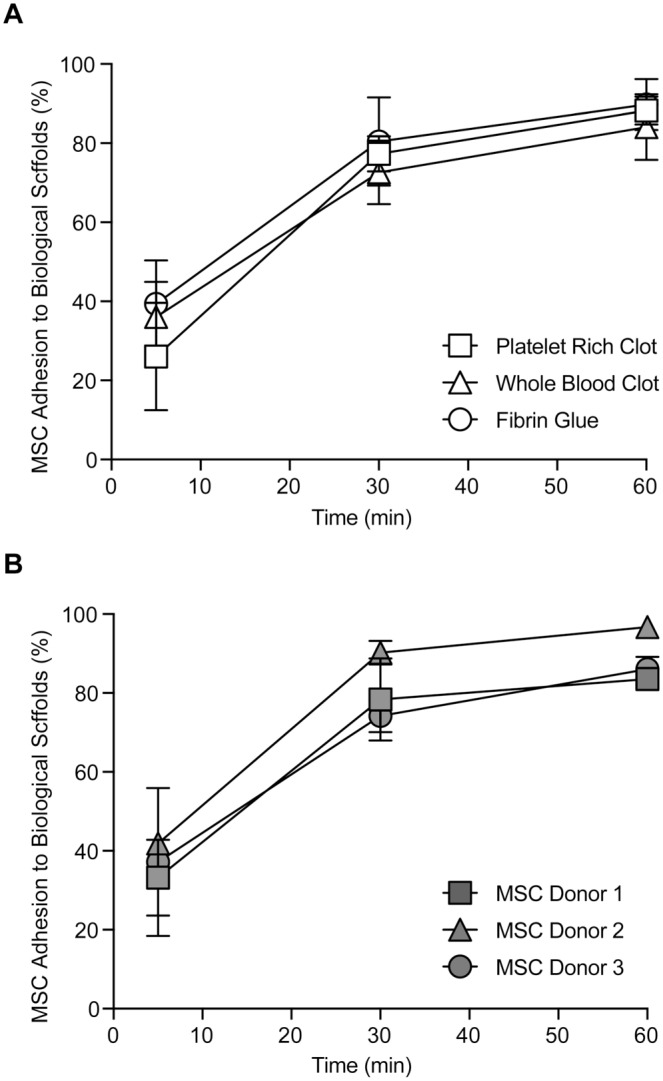
Mobilized mesenchymal stem cells (MSCs) rapidly adhere to a range of biological scaffolds. (A) Mobilized MSCs from a single donor show a rapid ability to adhere to a range of relevant biological scaffolds such as platelet-rich plasma and whole blood clots as well as fibrin glue. (B) Mobilized MSCs from multiple donors all exhibit the same rapid adhesion to a fibrin glue scaffold (n = 3).

Finally, having shown how MSC numbers can be reliably increased intraoperatively from the joint synovium and that Sm-MSCs fulfill the requirement of an MSC, we sought to determine whether these cells could be targeted to sites of interest via adhesion to relevant biological scaffolds. In these experiments, the ability of mobilized MSCs to adhere to FG, PRP, or WB clots was evaluated in vitro. Sm-MSCs from a single donor began to adhere to the surface of all 3 scaffolds within 5 minutes (Figure 4A). By 60 minutes, 89.7% (±6.5%), 88.3% (±3.5%), and 84.0% (±8.3%) of Sm-MSCs had adhered to the FG, PRP, and WB clots, respectively. To test donor variability, Sm-MSCs from 3 donors were allowed to adhere to FG scaffolds alone (Figure 4B). Sm-MSCs adhered rapidly, with little variation between donors. After 60 minutes, 83.5% (±2.6%), 96.7% (±1.0%), and 86.1% (±3.1%) of Sm-MSCs from each donor were adhered to FG scaffold.

## Discussion

Better cartilage and joint repair strategies are increasingly needed, and MSCs show great promise. To fully exploit these cells, new strategies and methods that limit operative time, cost, and tissue manipulation are required. Emerging data from several randomized controlled trials indicate that MSC therapy shows promise, at least in the short term.^31,48^ However, the majority of trials so far have used culture-expanded MSCs from bone marrow or adipose tissue or other ex vivo manipulation strategies. Here we provide proof of concept that minimally manipulated MSCs within the synovium can be easily mobilized and their numbers greatly augmented in a simple, single-stage procedure as part of knee arthroscopy. Furthermore, since these MSCs are derived from the synovium, which is the most potent source of chondrogenic progenitors, this technique has promising potential to increase the reparative capacity of the knee.^39^

The discovery of joint resident MSCs highlights potential new avenues to explore in the treatment of knee intra-articular pathologies.^30^ Studies have shown that numbers of MSCs in SF increase in patients with OA and cartilage injury^18,19,41^ and after ligament and meniscal damage.^28,33^ However, joint irrigation during arthroscopy, which aids visualization of the joint and removes debris, also removes SF with the potentially undesirable effect of reducing numbers of SF-MSCs.^12^ SF-MSCs are thought to derive from the synovium owing to their superior clonogenic and chondrogenic potential and shared gene expression profiles.^3,39,41^ The synovium itself holds great potential in providing an abundant endogenous supply of joint-specific MSCs; synovial MSCs are found at a frequency of ~1%, 500 times more numerous than in bone marrow.^10,13,17,39,43^ Additionally, the synovium, cartilage, and other joint structures share a common progenitor that is distinct from that of bone tissue,^26,27^ suggesting that the synovium harbors a joint tissue–specific stem cell population.^15,30,38^ The remarkable cartilage repair seen in patients with OA who are treated with knee joint distraction is testament to the intrinsic regenerative potential of these joint resident cells.^42^ In these patients, significant areas of denuded bone are replaced by intrinsic cartilage repair activity, providing functional and clinical benefits.^14,42,47^ It is thought that the temporarily altered biochemical and biomechanical environment created by joint distraction favors endogenous joint resident MSC activity and is in part responsible for this intrinsic repair where no extrinsic growth factors, scaffolds, or MSCs have been introduced.^4^

Synovial MSCs have already been used clinically with promising results; however, these studies also required a 2-stage procedure and in vitro culture expansion.^1,40^ The purpose of the current study was therefore to demonstrate proof of concept for a new, single-stage procedure to increase stem cell numbers in the joint, thereby avoiding ex vivo manipulation of tissue and cells. Furthermore, our data show that the Sm-MSCs rapidly adhere to biological scaffolds such as FG, PRP, and WB clots, thus demonstrating relevance in the context of existing procedures such as microfracture (Figure 1) or future practical applications with fibrin-based scaffolds.

Characterization of Sm-MSCs showed consistent phenotype and trilineage differentiation with their donor-matched SF counterparts, effectively demonstrating successful mobilization of joint resident MSCs. In the context of arthroscopic surgery, where saline inflation and irrigation of the joint result in the loss of SF-MSCs, this technique (when performed with the STEM device) increases the number and availability of stem cells (compared with those resident before and during arthroscopy); further, if left in the joint, these cells occupy a more suitable biochemical environment, being free from high-molecular-weight hyaluronan, which can limit their interaction with cartilage.^4^ Depending on the arthroscopic procedure used, Sm-MSCs could be aspirated from the joint for loading onto a scaffold or entrapped in the joint as a final-stage procedure.

One of the concerns of stem cell–based cartilage repair is cartilage hypertrophy, which leads to uneven articular surface. Synovium-derived MSCs exhibit superior chondrogenic capacity with limited potential to hypertrophy compared with MSCs derived from adipose and bone marrow.^15^ Our results using the STEM device are encouraging, supporting the potential of this technique and the synovium as a robust and convenient source of MSCs. Although colony numbers expressed here are low in comparison with the number of chondrocytes or MSCs currently used in the clinical setting, these Sm-MSCs have lost none of their proliferative or differentiation capacity as a result of culture expansion.^7,16^ Direct comparisons between these colony numbers and those obtained from fresh bone marrow aspirate are difficult owing to variations in aspiration technique. However, when an optimized aspiration procedure is used, in which small draw volumes are taken from multiple sites, this would be equivalent to up to 80 mL of bone marrow.^8,13^ For nonoptimized bone marrow aspirates in which larger volumes (~60 mL) are drawn before being concentrated,^11^ we can equate the number of colonies mobilized with the STEM device as being equivalent to up to 380 mL. More research and clinical studies are required to determine whether these Sm-MSCs will lead to clinically significant results. However, the idea is to achieve cartilage repair with a single procedure and thus avoid the need for 2-stage harvest and cell expansion. This will in turn reduce the burden and risks to the patient and the cost to the health care provider.

### Limitations

We recognize that synovial MSC mobilization was performed on a limited number of patients with a range of injuries. These injuries and the time between injury and surgery may affect the numbers of MSCs and the ability to mobilize them into the joint cavity. The primary purpose of the study was to determine to what extent synovial MSCs could be mobilized during a range of routine arthroscopies. In this study, the stem cells were removed from the joint for laboratory analysis, and so there was no expected benefit to the patient.

## Conclusion

Here we show proof of concept that a potent source of joint-specific mesenchymal stem cells can be accessed and those cells can be mobilized during routine arthroscopy. This technique can reliably and rapidly repopulate the knee with synovial MSCs that are viable, with trilineage potential in a single-stage procedure. Further studies are needed to ascertain whether this leads to better joint repair across the spectrum of arthroscopic procedures for damaged cartilage and other joint structures.
